# Supplementary material for: Stabilizing persistent currents in an atomtronic Josephson junction necklace
Source: Nat Commun. 2024 Jun 6;15:4831. doi: 10.1038/s41467-024-47759-7 (PMC11156925; doi:10.1038/s41467-024-47759-7)
Supplement: Supplementary file 1 — Supplementary Information [file 41467_2024_47759_MOESM1_ESM.pdf]

Stabilizing persistent currents in an atomtronic Josephson junction necklace: Supplementary Information

L. Pezzè,[1,2,3,*] K. Xhani,[1,2,3,*] C. Daix,[2,4,*] N. Grani,[1,2,4] B. Donelli,[1,5,3]
F. Scazza,[6,1,2] D. Hernandez-Rajkov,[1,2] W. J. Kwon,[7] G. Del Pace,[2,4] and G. Roati[1,2]

[1]*Istituto Nazionale di Ottica del Consiglio Nazionale delle Ricerche (CNR-INO), Largo Enrico Fermi 6, 50125 Firenze, Italy*
[2]*European Laboratory for Nonlinear Spectroscopy (LENS), Via N. Carrara 1, 50019 Sesto Fiorentino, Italy*
[3]*QSTAR, Largo Enrico Fermi 2, 50125 Firenze, Italy*
[4]*University of Florence, Physics Department, Via Sansone 1, 50019 Sesto Fiorentino, Italy*
[5]*University of Naples "Federico II", Via Cinthia 21, 80126 Napoli, Italy*
[6]*University of Trieste, Physics Department, Via A. Valerio 2, 34127 Trieste, Italy*
[7]*Department of Physics, Ulsan National Institute of Science and Technology (UNIST), Ulsan 44919, Republic of Korea*

Results of 3D numerical simulations. We first find the system ground state by solving the GPE by imaginary time evolution and in the presence of n barriers. We then instantaneously imprint a current of winding w_0 by multiplying the ground state wavefunction by the phase factor $\exp(-i2\pi w_0\theta)$, where θ is the azimuthal angle. We finally study the system dynamics by solving the time-dependent GPE. For a particle number $N = 6.8\times10^3$ (corresponding to the experimental condensate number), we obtain $\mu = 1.09$ kHz leading to a value of the healing length $\xi = 0.59\,\mu$m. Equation (11) (Methods) is solved numerically by the Fourier split-step method on a Cartesian grid of $\{N_x, N_y, N_z\} = \{256, 256, 80\}$ points dividing a grid size of length $-34.846\,\mu m \leq r \leq 34.846\,\mu m$ and $-11.0\,\mu m \leq z \leq 11.0\,\mu m$ in the radial plane and axial direction, respectively. The time step is set to $\Delta t = 1\times10^{-5}\,\omega_\perp^{-1}$. We characterise the condensate dynamics by studying the winding number, $w(t)$ calculated at at $z = 0$ and averaged over closed circle paths ranging from the inner to the outer radius.

Stable configuration. As discussed in the main text, for a fixed initial circulation w_0 we find the transition from unstable (decaying $w(t)$) to stable current (time-independent evolution of the winding) when the number of barriers n exceeds a critical value $n_c(w_0)$, see Fig. 1(a). In the stable configuration, 3D simulations reproduce the typical finding of the 1D case, namely that both the maximum of the superfluid speed and the phase gain at each junction decrease with n as shown in Fig. 1(b)-(c). In particular, figure 1(b) shows the absolute value of the superfluid velocity, $|\boldsymbol{v}(\boldsymbol{r}, t)|$, computed at the mean radius and for $z = 0$, as a function of the azimuthal angle θ. Figure 1(c) instead shows the time-averaged phase gain across each junction, $\delta\phi$, as a function of n (symbols), together with a $1/n$ fit (dashed lines). Finally, in Fig. 1(d), we plot the time-averaged $\delta\phi$ as a function of the time-averaged current (symbols). The dotted lines are the 1D current-phase relation obtained for the same number of junctions and rescaled to be consistent with the data. We see that the 3D results are consistent with the trend of an increasing critical current with n found in 1D.

Unstable configuration. If $n < n_c(w_0)$, we find that both w and the current decay in time via vortex emission. In Fig. 2 we show the numerical densities illustrating the microscopic mechanism of the vortex emission process. Vortices are emitted symmetrically from each barrier: they enter the ring from the central part, propagate along the transverse direction close to the barrier position until they enter the bulk and travel at the outer edge of the ring. Each vortex entering the bulk through the barrier causes a global decrease of the winding number by one. In particular, for the considered case of $n = 4$, the winding at $t = 5.7$ ms is equal to zero. We note that the detailed vortex emission process depends on the value of the barrier height considered.

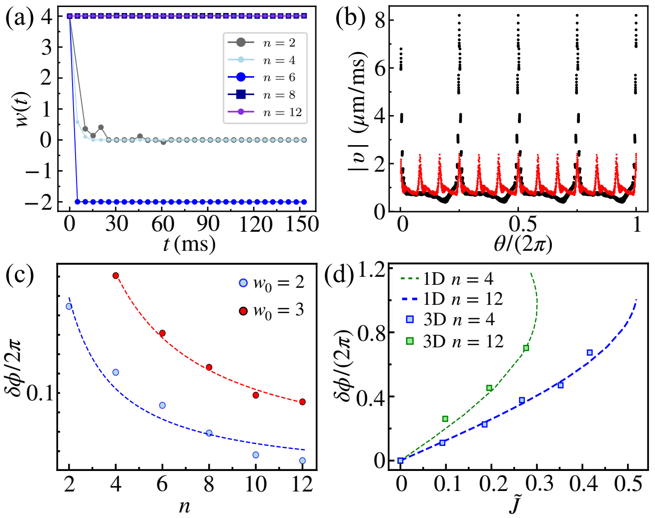

SUPP. FIG. 1. Results of the 3D GPE numerical simulations. (a) Winding number as a function of time for fixed $w_0 = 4$ and at different values of n (see legend). For the parameters considered in these simulations $n_c(w_0) = 8$. (b) Absolute value of the superfluid velocity extracted at the mean radius $R = 15\,\mu$m for $z = 0$, as a function of the azimuthal angle θ. Black (red) lines as obtained for $n = 4$ ($n = 12$), an initial winding number $w_0 = 2$ and at time $t = 5$ ms. (c) Time averaged phase gain across each junctions as a function of the number of barriers n and for different values of w_0. The dashed line is the fit function that goes as $1/n$. (d) Time-averaged phase-current values extracted from the 3D time-dependent GPE simulations for $n = 4$ (green squares) and $n = 12$ (blue). The dashed lines are the corresponding phase-current curves, obtained with 1D simulations [as in the inset Fig. 2(a)].

* These authors contributed equally to this work

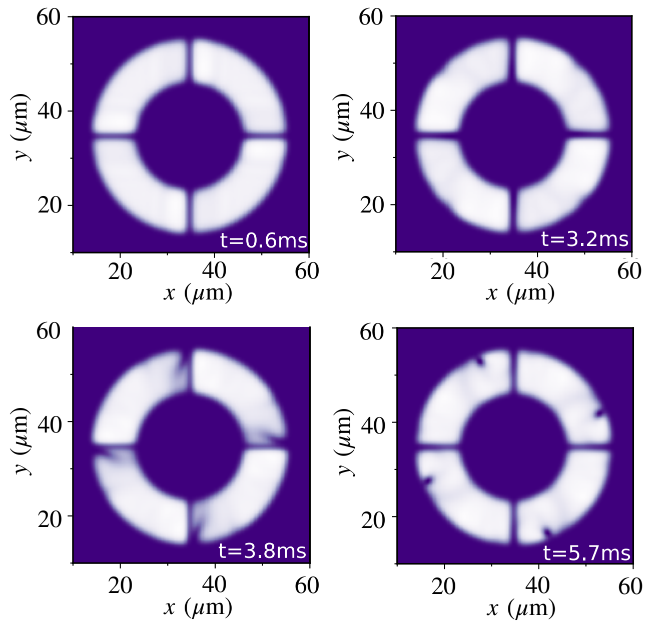

SUPP. FIG. 2. Density profiles obtained for an unstable configuration of the system ($w_0 = 4$, $n = 4$ and barrier height $V_0/\mu = 1.4$) from 3D GPE numerical simulations. Each panel represents the superfluid density integrated along the z-direction. The figure clearly shows the simultaneous nucleation of n vortices, each vortex being emitted from the junction edge.

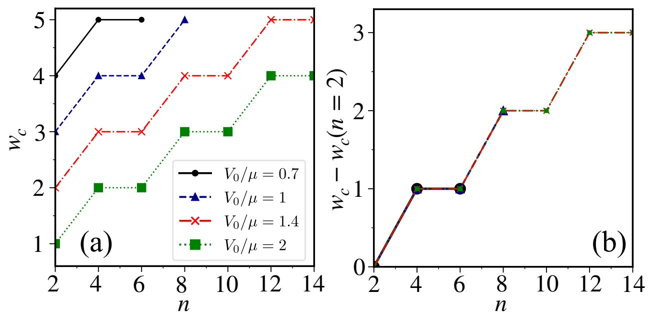

SUPP. FIG. 3. Effect of the barrier heights on the critical circulation. (a) Critical circulation $w_c(n)$ as a function of n and for different values of the barrier height V_0/μ. (b) By substracting from $w_c(n)$ the critical value for $n = 2$, the various curves collapse onto a single curve and we obtain a curve independent of V_0/μ.

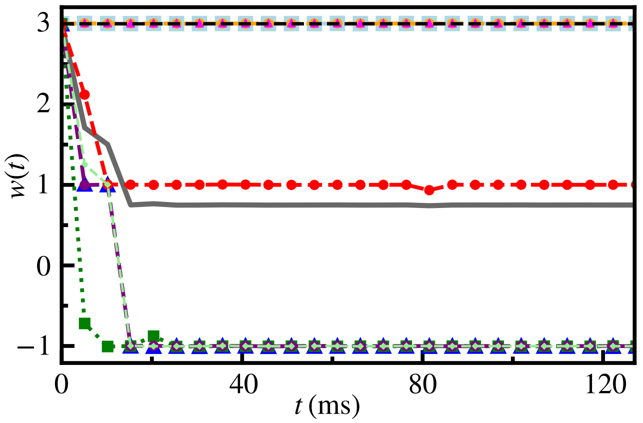

SUPP. FIG. 4. Winding number as a function of time for eight different simulations run with different barrier configurations (symbols with guides to the eye) and their average value (grey line). In each run, each barrier height and width is chosen randomly in the range $V_0/\mu_0 = [1.2, 1.6]$ and $\sigma = [0.68, 0.92]$ μm, respectively. The black dashed line show the stable winding number in the absence of noise, i.e. for identical barriers of height $V_0/\mu_0 = 1.4$ and width $\sigma = 0.8$ μm. The data are produced from 3D numerical simulations in the case of $n = 4$ barriers and at fixed $w_0 = 3$.

Critical circulation. The stability phase diagram can be either characterized by the critical number of barriers $n_c(w_0)$ for fixed w_0, as discussed above, or by the critical circulation $w_c(n)$. For a given n, the critical circulation is the largest value of w_0 for which we find a stable dynamics. In Fig. 3 we plot $w_c(n)$ as a function of n and for different values of V_0/μ. Interestingly, all the curves are parallel to each other and thus collapse on the same curve upon rescaling by w_c. In particular, by increasing V_0/μ the value of the critical circulation is shifted downwards. This is in agreement with the results in Ref. [1] for a single defect, which found that w_c becomes smaller at larger values of V_0/μ. The dashed white line in Fig. 4(b) of the main text is obtained by using V_0/μ as fitting parameter. We obtain that experimental data are well reproduced by $V_0/\mu = 1.80 \pm 0.05$. This value is larger than the experimental estimation of $V_0/\mu = 1.3$, even after taking into account a decrease by 15% of the chemical potential due to particle losses. The reason behind this discrepancy is investigated in the following paragraph.

We finally notice that the increase of $w_c(n)$ with n shows no qualitative change when the barrier height becomes lower than the chemical potential (weak link regime). However, clearly observing the transition from unstable to stable current states as a function of n for $V_0 < \mu$ would require the preparation of initial states with circulations much larger than those considered in this work.

Effect of non-identical barriers on the current's stability. Due to the finite resolution of the DMD-created optical potentials, the experimental barriers creating the JJN are not identical, but their height and size is distributed around the mean values mentioned in the main. To check whether non-identical barriers could affect the current stability in the JJN, we study this case with 3D numerical simulations. In particular, in Fig. 4 we report the time evolution of the winding for $n = 4$ barriers and $w_0 = 3$, which is stable for identical barriers, when each barrier height and width are randomly selected from Gaussian distributions of mean values $V_0/\mu_0 = 1.4$ and $\sigma = 0.8\,\mu$m and standard deviation $\Delta V_0/\mu_0 = 0.2$ and $\Delta\sigma = 0.12$ μm, respectively. The mean and standard deviation values correspond to the ones measured from the experimental characterization of the barriers. In the figure, we show the winding number as a function of time for 8 different runs (symbols), corresponding to different configurations of the barriers. We also plot the statistical mean value (solid line). These simulations reproduce qualitatively the experi-

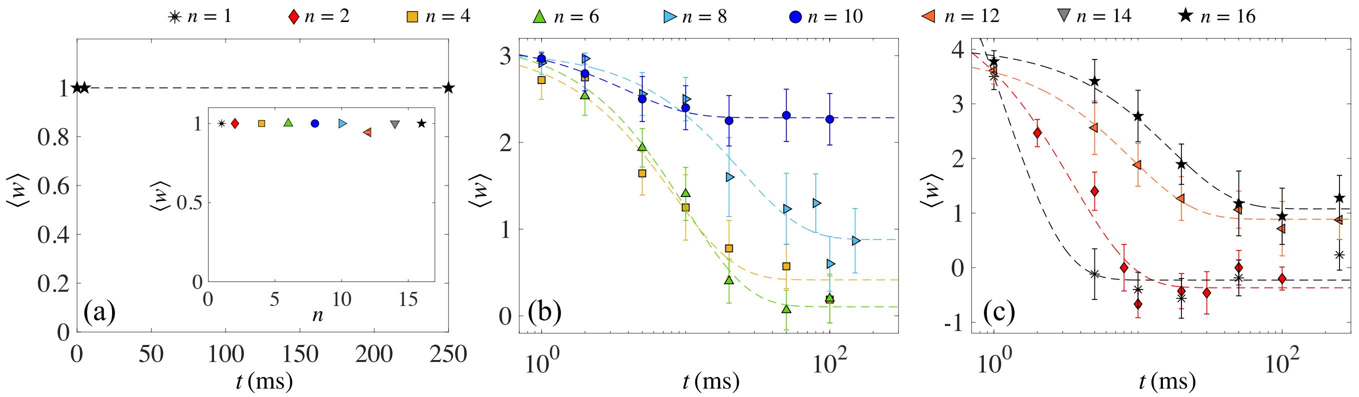

SUPP. FIG. 5. Statistically-averaged winding number as a function of time and for different n (symbols). The different panels refer to different values of w_0: $w_0 = 1$ (a), $w_0 = 3$ (b) and $w_0 = 4$ (c). Dashed lines represent the exponential fit of each dataset, using the same fitting function as in Fig. 4(a). The inset of panel (a) reports the averaged winding number at $t = 250$ ms as a function of n for $w_0 = 1$.

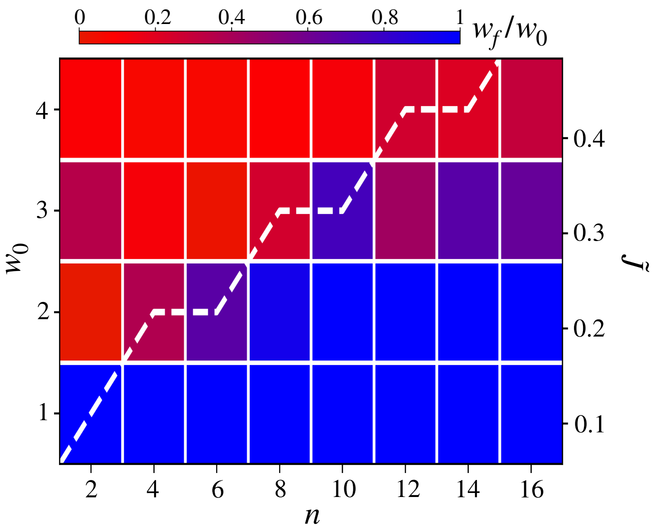

SUPP. FIG. 6. Measured ratio w_f/w_0 (colormap) as a function of w_0 and n. The white dashed line is the same as in Fig. 4(b).

mental findings: for some barrier configurations, the winding number remains constant in time, for some others it decays, eventually also reaching negative values. Correspondingly, also the average winding number (solid line) decays in time. To summarize, having non-identical barriers is observed to reduce the stability of currents in the JJN for the same w_0, explaining the discrepancy in V_0/μ found between the experimental and numerical phase diagram.

Rate of thermally- and quantum-activated decay processes. The energy barrier separating the $w = 0$ from the $w = \pm 1$ states can be estimated as twice the Josephson energy (see e.g. [2]), linearly dependent to the critical current. Following Refs. [3, 4], we estimate a thermally-activated decay rate $\nu_{th} \simeq 0.54$ Hz for the experimental temperature $T = 60$ nK (at which the condensate fraction is 80%), i.e., a phase slippage time of 1.84 s. We have checked numerically – by solving the collisionless Zaremba-Nikuni-Griffin model [5, 6] at the experimental temperature – that finite-temperature dissipation does not affect the critical winding number ($w_0 = w_c$) for at least 150 ms and marginally affects the decay time. Furthermore, by following Ref. [3], we estimate a typical quantum tunnelling-induced decay time of the order of hundreds of seconds. Note that both thermal and quantum phase slippage times further increase with the critical current and therefore with the number of junctions.

Experimental phase profile in the JJN. As already commented in the main text, the interferograms associated with stable realizations, namely in which $w = w_0$, show interference fringes with a clear polygonal structure (e.g. squared for $n = 4$), which are a manifestation of the phase jump at each Josephson junction. Thanks to the high resolution of the imaging setup, we can extract the local relative phase between the ring and the reference central disc, ϕ, as a function of the azimuthal angle θ, as reported in Fig. 7. The interferograms in polar coordinates [(a)] display a characteristic step-like shape of the fringes, closely resembling the predicted behavior of the JJN phase by the analytical model and from the numerical simulations (see the inset of Fig. 2(a) for comparison). We then quantitatively extract the value of $\phi(\theta)$ as the phase shift in the sinusoidal fit of a slice of the polar interferogram at constant θ. As shown in Fig. 7 (b), the $\phi(\theta)$ trend clearly deviates from the linear behavior expected in a clean ring [7], but it rather exhibits a number of jumps in correspondence of the barriers in the JJN.

Experimental stability phase diagram. In Fig. 5 we provide additional experimental data regarding the statistically-averaged winding number as a function of time and for different n and w_0. In the case $w_0 = 1$, $\langle w \rangle$ is found to be constant in time up to 250 ms for any n. In particular, in Fig. 5(a) we plot the case $n = 16$, averaged over about 20 realizations. The inset of Fig. 5(a) shows $\langle w \rangle$ at time $t = 250$ ms and for n ranging from 1 to 16. Only in the case $n = 12$, we found a single experimental realization (out of 18 independent runs) with $w = 0$. In Fig. 5(b) and (c) we plot the cases $w_0 = 3$ and $w_0 = 4$ [the case $w_0 = 2$ is shown in Fig. 4(a)].

In Fig. 6 we report the analogue of the stability phase diagram of Fig. 4(a) here plotting w_f/w_0, where w_f is the average circulation at long time, as obtained from a fit (see main text).

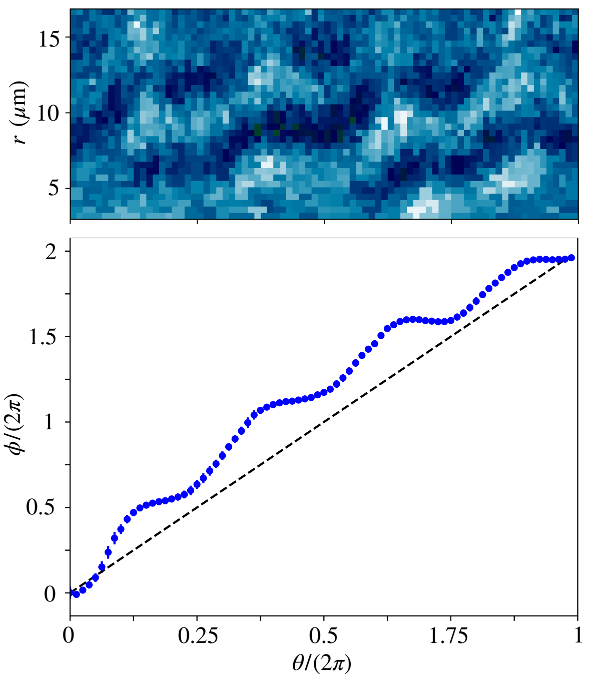

SUPP. FIG. 7. Extraction of the azimuthal profile of the JJN phase from the experimental interferograms. The upper panel shows the interferogram image unwrapped into polar coordinates. A sinusoidal fit of each azimuthal slice is performed by using the function $A\cos(\alpha r + \phi)$. To enhance the fringe contrast, for each value of θ we consider a radial slice of the polar interferogram averaged over $\Delta\theta = 0.47$ rad. The image is obtained by averaging 5 similar experimental spiral patterns and unwrapping the resulting image. The lower panel is the fitted azimuthal trend of ϕ, showing a phase jump in correspondence of each barrier. The unwrapped phase here is averaged over the profiles of all experimental images for ($w_0 = 2$, $n = 4$) where $w(t) = w_0$ at times $t > 10$ ms.

This shows the experimental deterministic realization of stable circulation states for $w = 1$ and $w = 2$ in a toroidal trap with up to $n = 16$ junctions.

Superfluid fraction and the $f(\tilde{w}, n)$ function. The superfluid fraction for neutral atoms in a ring trap rotating at an angular velocity Ω (pointing along the axis z perpendicular to the ring) can be defined as [8, 9]

$$f_s = 1 - \lim_{\Omega\to 0} \frac{L_z}{I_{\rm cl}\Omega}, \qquad \text{(S.1)}$$

where $\boldsymbol{L} = \int d\boldsymbol{r}\, m|\psi(\boldsymbol{r})|^2 \boldsymbol{r} \times \boldsymbol{v}(\boldsymbol{r})$ is the expectation value of the angular momentum, $I_{\rm cl} = \int d\boldsymbol{r}\, mr^2|\psi(\boldsymbol{r})|^2$ is the classical moment of inertia, and the superfluid state with $w_0 = 0$ is considered.

In a 1D ring of radius R, $I_{\rm cl} = mR^2$ and $L_z = mR\int d\theta\rho(\theta)v(\theta)$, where $\rho(\theta)$ and $v(\theta)$ are the density and the velocity along the ring. Equation (S.1) becomes $f_s = -2\pi \lim_{\Omega\to 0} \frac{J}{\Omega}$. Replacing into this equation the expression for $J/\Omega_{\rm R}$ given in Eq. 8 (see Methods), we find $f_s = f(\tilde{w}, n) = (2\pi)^2(\int_0^{2\pi} d\theta \frac{1}{\rho(\theta)})^{-1}$. Restricting the integration to a unit cell of size d (corresponding to the angular distance between two junctions) as in Refs. [8, 9], and normalizing $\rho(\theta)$ to 1 within that cell, we recover Leggett's equation,

$$f_s = \frac{1}{\frac{1}{d^2}\int_{\rm cell} \frac{d\theta}{\rho(\theta)}}. \qquad \text{(S.2)}$$

Equation (S.2) can be also derived by noticing that the two bounds in Eq. (6) coincide in 1D. In Fig. 8(a) we plot f_s (circles) and f_c [corresponding to $f(\tilde{w} = \tilde{w}_c, n)$, dots] as a function of n. Both functions decrease with n until the barriers start to overlap. In the homogeneous case ($n = 0$), we have $\rho(\theta) = 1/(2\pi)$, $f(\tilde{w}, 0) = 1$ and thus $f_s = 1$, while f_c is not definited. In Fig. 8(b) we plot $f(\tilde{w}, n)$ as a function of $\tilde{w}$ for $n = 6$.

It should be noticed that the variational bounds in Eq. (6) have been obtained by Leggett [8, 9] by considering a small phase twist in a narrow ring of relatively large radius. In our system, the superfluid fraction evaluated according to Eq. (S.1) may include additional transversal finite-size effects due to the non-negligible thickness of the ring.

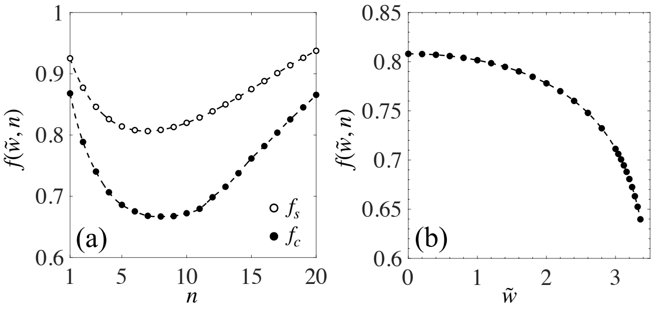

SUPP. FIG. 8. Function $f(\tilde{w}, n)$ calculated for stationary states of the 1D GPE. Panel (a) plots $f(\tilde{w}, n)$ as a function of n for two interesting cases: $f_c(n) = f(\tilde{w} = \tilde{w}_c, n)$ (dots dots) and $\lim_{w=0,\Omega\to 0} f(\tilde{w}, n) = f_s$ (circles), corresponding to Leggett's superfluid fraction, Eq. (S.2). Panel (b) shows $f(\tilde{w}, n)$ as a function of $\tilde{w}$ and for $n = 6$ (dots). In both panels, lines are guides to the eye.

[1] K. Xhani, G. D. Pace, F. Scazza, and G. Roati, "Decay of persistent currents in annular atomic superfluids," *Atoms* **11**, 109 (2023).

[2] K. C. Wright, R. B. Blakestad, C. J. Lobb, W. D. Phillips, and G. K. Campbell, "Driving phase slips in a superfluid atom circuit with a rotating weak link", *Phys. Rev. Lett.* **110**, 025302 (2013).

[3] B.I. Halperin, G. Refael and E. Demler, "Resistance in superconductors", *International Journal of Modern Physics B* **24**, 4039 (2010).

[4] A. Kumar, S. Eckel, F. Jendrzejewski, and G. K. Campbell, "Temperature-induced decay of persistent currents in a superfluid ultracold gas", *Phys. Rev. A* **95**, 021602(R) (2017).

[5] A. Griffin, T. Nikuni, and E. Zaremba, *Bose-Condensed Gases at Finite Temperatures* (Cambridge University Press, 2009).

[6] K. Xhani and N. P. Proukakis, “Dissipation in a finite-temperature atomic Josephson junction”, *Phys. Rev. Res.* **4** 033205 (2022).

[7] G. D. Pace, K. Xhani, A. M. Falconi, M. Fedrizzi, N. Grani, D. H. Rajkov, M. Inguscio, F. Scazza, W. Kwon, and G. Roati, “Imprinting persistent currents in tunable fermionic rings”, *Phys. Rev. X* **12**, 041037 (2022).

[8] A. J. Leggett, “Can a solid be ”superfluid”?”, *Phys. Rev. Lett.* **25**, 1543 (1970).

[9] A. J. Leggett, “On the superfluid fraction of an arbitrary many-body system at $T = 0$”, *J. Stat. Phys.* **93**, 927 (1998).

[10] W. J. Kwon, G. Del Pace, R. Panza, M. Inguscio, W. Zwerger, M. Zaccanti, F. Scazza, and G. Roati, “Strongly correlated superfluid order parameters from dc Josephson supercurrents”, *Science* **369**, 84 (2020).
